# Adolescent Addiction to Short Video Applications in the Mobile Internet Era

**DOI:** 10.3389/fpsyg.2022.893599

**Published:** 2022-05-10

**Authors:** Lihong Lu, Mei Liu, Binchao Ge, Zijin Bai, Ziqi Liu

**Affiliations:** ^1^College of Modern Economics and Management, Jiangxi University of Finance and Economics, Nanchang, China; ^2^School of Economics and Management, East China Jiaotong University, Nanchang, China; ^3^School of Communication and Design, Sun Yat-sen University, Guangzhou, China

**Keywords:** adolescent, addiction to short video applications, UGC, flow theory, immersive experience

## Abstract

The adolescent addiction to short video applications is becoming increasingly prominent, which has brought great challenges to the physical and mental health and daily life of the adolescents. This manuscript conducts an empirical study on the contributing factors of the adolescent addiction to short video applications based on the user generated content (UGC). In our study, 96 participants aged 15–25 were surveyed by questionnaire, and then cross-analysis of individual factors and SEM analysis of UGC content factors were carried out. Through the analysis of individual factors of the adolescent addiction from the perspective of gender, age, and family environment, this study reveals that male users are more addicted to the use of applications (APP), and such addiction varies with age, and prolonged family members’ use of short video APP can also exacerbate the adolescent addiction degree. Furthermore, through verification of the theoretical model, it indicates that UGC perception and the degree of boredom in daily life have a significant positive effect on the level of addiction to short video applications, and the degree of boredom in daily life plays a significant mediating role between them. Based on the research on the influences of UGC on the adolescent immersive experience, this study proposes a mechanism of the adolescent addiction to the use of short video applications in the mobile Internet age to provide a better service guarantee for the adolescents.

## Introduction

From the graphic age of forums and communities to the “micro” dissemination of Weibo and WeChat, and to the visual transformation, social media platforms have been iteratively evolving and information modes have become increasingly enriched with technological upgrades (Zhan and Zhou, 2018; Klein and Watson-Manheim, 2021; Wang et al., 2021a,b). In terms of the content curators, the user generated content (UGC) mode has become the mainstream of content production in social media (Taleb et al., 2015; Liu et al., 2021; Yi et al., 2021a), whose focus shifts from words, images and pictures to audio and video, and allows the users to display or provide their original content to other users through the Internet platform, reflecting the advantages of more affinity and personalization (Elgaaied-Gambier and Mandler, 2021). In the short video mode, the users absorb external information while filling out the platform content and assume the dual roles of online content curator and viewer (Dogruel et al., 2021), which realizes and makes the Internet contents varied, wide and specialized. The adolescents are becoming the main force in the mobile Internet and short video industries (Kaye et al., 2021), and the integration and intelligent application of life, learning and entertainment have become the trend of their use of social media (Qu, 2021). Especially when they are bored in daily life, the adolescents tend to pursue more experiential things, perceive novel stimuli of the UGC in short videos, and concentrate on and fully immerse themselves in them. This immersive experience, also known as flow experience, has also been applied in previous studies on online transactions (Sharif and Naghavi, 2021), and hypnosis (Bowers et al., 2018).

Short videos make the users excited through their novel contents and entertainment (Nam and Jung, 2021), while they may have negative effects in some cases. When browsing short videos gradually becomes the daily habit and lifestyle of the adolescents who are unable to restrain themselves from the UGC and forms a negative feedback effect on their daily life perception (Chung et al., 2021), it will aggravate the adolescent addiction to short video applications (apps for short) (Throuvala et al., 2019). Most research on the UGC is based on the promotion power and its influence of consumer preferences (Mohammad et al., 2020; Soylemez, 2021), and there is little discussion about the addiction of UGC perceived stimuli. To deeply explore the causes of short video application addiction among teenagers in the era of mobile Internet, individual adolescent factors and content factors of UGC are the internal and external factors that need to be investigated. Based on this, the study carried out cross-over analysis in part 1 to analyze the relationship between personal basic statistical characteristics and addiction to short video application concretely. In order to comb and analyze the mechanism performance of UGC content perception leading to short video application addiction in teenagers, empirical research was conducted in part 2. The organic combination of the two parts can further explain the logical process of adolescent addiction in the mobile Internet era, and can explain the law of addiction through the application of heart flow theory.

## Literature Review

### Concept Definition

#### Adolescents

In the conceptual definition of adolescents, adolescence is currently regarded by academics as a transitional stage between childhood and adulthood (Herting and Sowell, 2017), with significant staged performance of social and cognitive abilities (Arnett, 2000; Caballero et al., 2016), thus the current age span positioning of adolescent is derived from this. However, due to the different perspectives of scholars, the performance of age division is mostly different. For example, Enstad et al. (2019) divided adolescence into two stages, mid-adolescence (14–16 years) and late adolescence (17–25 years), based on a longitudinal study of the population, the Norwegian Tracking Opportunities and Issues Study, and the Australian International Youth Development Study. Orben et al. (2020) positioned adolescence from a mental health perspective as 10–24 years, analyzing how social deprivation during adolescence has far-reaching effects. Becker et al. (2021) positioned the age span of adolescence as 15–17 years based on the physical status of sleep changes before and after the COVID-19 epidemic in adolescents. Further, Odgers and Jensen (2020) identified the age region of adolescents as 13–18 years based on the state of mental health in the digital age. There is no clear standard for the age of adolescents in the academic community. Not only because of the different perspectives, but also because of the influence of the geographical environment in which the scholars live, or the inconsistent characteristics and growth patterns of the surrounding population. Therefore, Therefore, the definition of 15–25 years proposed by Leung et al. (2019) in combination with the World Health Organization seems to be more convincing, as it is not only the common result of global experts, but also includes the range proposed by most of the above-mentioned scholars as far as possible. In summary, this study concludes that the adolescents span can be identified as within the 15–25 years interval, an age when social skills are first formed and cognitive abilities begin to develop, an important time for humans to lay the foundation for future growth.

#### Addiction and Addiction to Short-Video Applications

Addiction is a state that fundamentally maintains a particular behavior (Heather, 2020). It is a subconscious phenomenon which excludes compulsion and other behavioral factors that are difficult for patients to get rid of (Heather, 2019). In recent years, short video applications have become an important area of digital content consumption. As an emerging entertainment method, the use of short video applications can stimulate users with novel content and entertainment (Nam and Jung, 2021). However, the stimulus can also be a hindrance (Zhang et al., 2019), leading to distraction, poor time management, and loss of learning time. They are all known addictions (Hong et al., 2014; Gao et al., 2017).

#### Boredom

As one of the most common emotions in daily life (Goetz et al., 2014), boredom has been widely concerned by the academic community. In the early stage, scholars tended to discuss the causes and process of boredom. O’hanlon (1981) defined boredom as a unique psychophysiological state with interrelated and inseparable emotion, motivation, perception, and cognitive consorts. Combined with the perspective of language habit, Mikulas and Vodanovich (1993) believed that boredom was a relatively low arousal state, which was attributed to insufficient stimulation. However, with the deepening of people’s cognition of the concept of boredom, people begin to realize that boredom is not a weak psychological state in most cases, but an “aversion state lacking but unable to engage in satisfying activities” (Weybright et al., 2020). Previous studies only considered boredom as a negative emotional experience, while they ignored its strong role in guiding human behavior, which could stimulate individuals to seek new experiences (Bench and Lench, 2019; Deng et al., 2020). Thus, boredom is considered as a “social plague” by current academic circles (Pekrun et al., 2010; Schwartze et al., 2021).

Currently, boredom can be divided into two types: state boredom and trait boredom (Vogel-Walcutt et al., 2012). Among them, state boredom is an emotion that appears in a specific situation. It will occur when a person experiences both the (objective) low arousal neural state and the (subjective) low arousal response of the depression, dissatisfaction, or disinterest. Therefore, state boredom is a transient emotional state (Vogel-Walcutt et al., 2012; Gana et al., 2019). Trait boredom, on the other hand, refers to the general lack of interest and the view that the environment is static, leading to disconnection with the environment. It is a personal tendency to feel bored and is considered as a long-term and chronic emotional tendency, which is related to various mental health conditions (Farmer and Sundberg, 1986; Gana et al., 2019). It should be noted that both state boredom and trait boredom are prevalent in current social development. “Immersive experience” was first proposed by Csikszentmihalyi and LeFevre (1989). It refers to the process in which people pay high attention to the current activities and fully engage in them when they are in a certain state of concentration, and then obtain satisfaction after completion (Csikszentmihalyi and Csikzentmihaly, 1990). At present, “immersive experience” is widely used in online games, virtual worlds, learning and work (Wan and Chiou, 2006; Ahmad and Abdulkarim, 2019; Rachmatullah et al., 2021). It is mostly reflected as a goal-oriented preference (Stavrou et al., 2015) and an organic expression of persistent perception (Marty-Dugas et al., 2021). At present, the emergence of UGC enriches people’s boring life. It satisfies people’s instinctive tendency for novel and complex feelings and experiences in the form of fresh content (Mikulas and Vodanovich, 1993). People’s attention gradually forms from immediate perception to sustained attention (Rajamma et al., 2019; Geng and Chen, 2021). UGC perceptual stimulation is also gradually transformed from instant experience to UGC immersive experience.

### Addiction Stimulated by User Generated Content Perception

As the mobile Internet develops, the content and form of social media are no longer limited to traditional media, and the user generated content (UGC for short), an emerging online information resource creation and organization mode, has become popular. Through this mode, the users can share their own contents, such as texts, pictures, and videos (Krumm et al., 2008; Yi et al., 2020), which in turn promotes the diversity of the UGC and enhances the sense of novelty of the users. The UGC has been widely applied in many industries. Therefore, the academia has put forward the concept of the UGC perception, namely, the perception and feeling of the public when watching user-created texts, pictures, videos, and has conducted in-depth research on them. For example, in terms of social connectivity, scholars have concluded through research that the UGC perception can enhance social connectivity by stimulating the users to recommend the UGC to friends to expand the range of users (Li et al., 2012; Yi et al., 2021b). While differentiated UGC will exert different effects on different products (Estrella-Ramón and Ellis-Chadwick, 2017), studies have attempted to analyze the influences of personality traits on the flow state experience and the types of use of the UGC and have found that the use of UGC for entertainment is positively associated with extraversion, while flow experience increases the propensity of the use of the UGC for entertainment (Moon et al., 2014). In terms of marketing, the interaction between UGC sources and content sponsorship will affect consumers’ brand preference (Kim and Lee, 2017; Li X. et al., 2021), and related service management will also exert an impact on UGC reviews (Perez-Aranda et al., 2018). Also, some scholars have applied UGC to digital content consumption (Zhao et al., 2022), telecom payment (Safavi et al., 2019), and brand competition (Liu et al., 2019). For the adolescent group, the richness and variety of UGC and the sense of freshness it brings may stimulate them to keep using the short video apps so much to an addictive state. Therefore, this study proposed the following hypothesis:

*H1: UGC perception has a significant positive effect on the addictive use of short video apps.*

### Addiction Triggered by Boredom in Daily Life

At some point in everyone’s life there is an experience of being bored. They felt empty and apathetic because they felt every action led to boredom (Bargdill, 2000). Meanwhile, research points to boredom as a common experience that affects people on multiple levels, including their thoughts, feelings, motivations, and actions (Chou et al., 2018). Studies have found that boredom will stimulate individuals to seek novel experiences (Bench and Lench, 2019; Deng et al., 2020). Some scholars have explored the psychological impacts of state boredom in Chinese adults during the COVID-19 outbreak, finding that the aggravation of state boredom promote the individuals’ use of Internet media (Chao et al., 2020). As a new entertainment means of Internet media, the short video integrates texts, sound, and pictures. It has rich and diverse contents and can meet various entertainment demands of the public. The individuals also use short video apps to alleviate their boredom of daily life (Jaffar et al., 2019). However, almost all activities in daily life may lead to true addiction (Billieux et al., 2015), and many factors such as personality traits and family environment may also cause Internet addiction (Weinstein and Lejoyeux, 2010; Monacis et al., 2021). In studies on the relationship between harmful use of alcohols and Internet addiction among college students, scholars found that Internet addiction was related to harmful use of alcohols among college students, and pleasure-seeking as a common feature of these two behaviors played a role in promoting the relationship between them (Yen et al., 2009). In light of previous research, scholars such as Guillot et al. (2016) found that the Internet addictive behaviors of the would-be adults are related to anhedonia. Given that short video apps are based on the Internet and that boredom proneness is positively related to Internet addiction (Chou et al., 2018; Martínez-Vispo et al., 2019), boredom proneness may also be a good predictor of smartphone addiction. According to the survey, boredom and feeling seeking reach the peak in adolescence (Spaeth et al., 2015; Freund et al., 2021), and there is a significant positive correlation between loneliness and mobile phone addiction, as well as between boredom propensity and mobile phone addiction (Li N. et al., 2021). Some scholars explored the relationship between boredom and adolescent use of Facebook and found that when the adolescents are more sensitive to boredom, they are more likely to overuse Facebook and become addicted to it (Donati et al., 2022). Given that, this study proposed the following hypothesis:

*H2: The degree of boredom in daily life has a significant positive effect on the addictive use of short video apps.*

### Boredom in Daily Life Exacerbated by User Generated Content Perception

Studies have found that in recent years, the adolescents’ sense of boredom has been increasing, which is likely to result from environmental factors and their own development (Weybright et al., 2020). For adolescents, the deepening of daily boredom will inevitably affect their learning, mental health (Schwartze et al., 2021). Rich in content, The UGC requires a low threshold for creation and has diversified creation styles and types. Compared with daily paper reading and other experience means, the UGC can meet the entertainment needs of different groups of individuals in multiple ways to attract the public, including the young adolescent group. UGC on the internet is not merely used for entertainment or passing time but may also weakens the boredom of daily life (Poon and Leung, 2013). However, scholars also pointed out that the richness of short video contents has intensified individuals’ addiction to short videos to some extent (Yang et al., 2021), thereby reducing individuals’ interest in the surrounding things in daily life. Nowadays, many individuals choose to use mobile phones for temporary entertainment after work or study to reduce fatigue and boredom. Scholars have also conducted related research on whether individuals will continue to work, study or rest after a temporary break. Fernandez et al. (2020) explored the effect of short-term withdrawal on potential behavioral addiction and found that after withdrawal, the respondents showed symptoms such as craving and relapse, and they were less interested in things around them; Dora et al. (2020) expounded that the participants were more likely to use their smartphones when they were bored or tired and would feel more tired and bored after putting down their smartphones. Thus, this study proposed the following hypothesis:

*H3: The UGC perception has a significant positive effect on the degree of boredom in daily life.*

### User Generated Content Perception as a Highly Stimulating Immersive Experience

“Immersive experience” was first proposed by Csikszentmihalyi and LeFevre (1989). It refers to the process in which people pay high attention to the current activities and fully engage in them when they are in a certain state of concentration, and then obtain satisfaction after completion (Csikszentmihalyi and Csikzentmihaly, 1990). At present, “immersive experience” is widely used in online games, virtual worlds, learning and work (Wan and Chiou, 2006; Ahmad and Abdulkarim, 2019; Rachmatullah et al., 2021). It is mostly reflected as a goal-oriented preference (Stavrou et al., 2015) and an organic expression of persistent perception (Marty-Dugas et al., 2021). At present, the emergence of UGC enriches people’s boring life. It satisfies people’s instinctive tendency for novel and complex feelings and experiences in the form of fresh content (Mikulas and Vodanovich, 1993). People’s attention gradually forms from immediate perception to sustained attention (Rajamma et al., 2019; Geng and Chen, 2021). UGC perceptual stimulation is also gradually transformed from instant experience to UGC immersive experience; and on this basis, some scholars have found that boredom plays a mediating role in social media (Bai et al., 2021). In view of the above analysis, this study argued that the adolescents have limited choices of entertainment in their daily life, whereas the UGC is rich, diverse and fresh, and compared to the stimulus brought by the boredom in daily life to the adolescents, the UGC perception will trigger a higher stimulus to the adolescents and the flow experience effect will be more apparent, thereby reducing the interest of the adolescents in the surrounding things, aggravating the boredom of their daily life and relying on the use of short video apps. Therefore, this study proposed the following hypothesis:

*H4: The degree of boredom in daily life has a significant mediating effect between UGC perception and addictive use of short video apps.*

**FIGURE 1:**
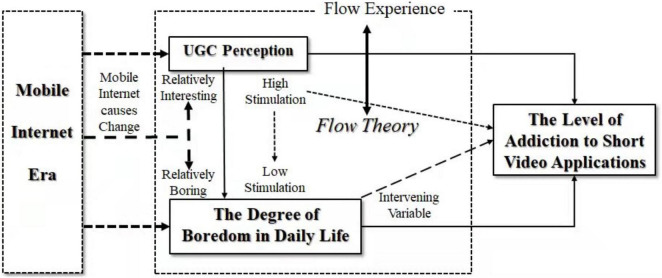
Theoretical model of adolescent addiction to short video apps.

Considering the above four hypotheses, the theoretical model in this study is constructed as shown in Figure 1.

## Research Design

### Participants

To ensure the stability of the test environment and eliminate the interference of the participants’ surrounding environment, this study adopted an experimental method to analyze the participants from universities and newly employed individuals in Nanchang City, Jiangxi Province, China, to explore the contributing factors of the adolescents’ addictive use of short video apps in the mobile Internet age. Following the principles of purpose, simplicity, clarity and logic of the questionnaire design, 134 questionnaires were distributed in this study, and a total of 103 questionnaires were recovered, with an effective recovery rate of 76.87%. In light of the age range of the adolescents defined by the World Health Organization, the participants aged 15–25 (including 15 and 25 years old) were finally screened, and 96 questionnaires were obtained, and they were divided into three groups according to the age range, namely, the first group ranged from 15 to 17 years old, the second group 18–21 years old, and the third group 22–25 years old.

### Process Design

This study attempted to collect data by distributing questionnaires online to the adolescents to avoid inaccurate data caused by mutual influences. It divided the respondents into groups based on their ages after eliminating invalid questionnaires with random answers, missing questions and inconsistency. Given that, this study carried out the following two explorations: Part 1 aimed to explore the individual factors of the adolescent addiction, that is, based on the information obtained from the Internet and personal experience, the adolescent addictive use of short video apps was analyzed from the aspects of gender, age and family environment; Part 2 aimed to explore the adolescent addiction from the factor of the UGC and verify the proposed theoretical model, that is, applying Smart-PLS to conduct the empirical analysis to verify the hypotheses proposed in this study and construct the mechanism of the adolescent addictive use of short video apps in the mobile Internet era.

### Scale Selection

This study adopted the immersive experience scale developed by Rigby et al. (2019) to measure the UGC perception. This scale can be used to evaluate individuals’ immersive experience in different situations, with an internal consistency of 0.859 and excellent applicability. It has been applied in many fields such as medicine, education, and psychology. We noticed that the respondents who participated in the questionnaire were students from a university in the United Kingdom, and they received credits for it. So, we think it has applicability for teens. In this manuscript, it was used to assess the immersive feelings of the adolescent when using Douyin through the following five items, example item as “How much does Douyin account for in your life?” And items were named UG1, UG2, UG3, UG4, UG5, and were scored based on a five-point scale to further explore the relationship between the UGC perception and adolescent addictive exposure to short videos.

The degree of boredom in daily life was measured by the User Engagement Scale adopted in the study by O’Brien and Cairns (2015). With high reliability and validity, the scale was used for self-report measures in education and multimedia fields in the early days (Jacques, 1996). Its dimensions involve boredom, attention, control, motivation, and patience. It has been gradually applied to multiple fields such as social networks, games. The survey was conducted in a university to investigate stories that students are willing to share in social occasions and analyze their user experience of online news. Therefore, we believe that the scale is suitable for the study of adolescents. In this study, the two dimensions of sustainability and concentration were selected, and the items were revised and designed in combination with the research themes, comprising three items, and the example item as “Daily life did not go as I planned.” These items were named RC1, RC2, and RC3, respectively, and scored based on a five-point scale to measure the boredom of adolescents’ daily life, which is beneficial to further understand the causes of the adolescents’ addiction and addiction mechanism.

This manuscript measured the addictive use of short video apps by adopting the addiction scale designed by Smith and Short (2022) who believed that social media addictive symptoms can affect adolescents’ emotions, cognition, mental health, etc. and measured individuals’ degree of addiction through the social media addiction scale whose reliability reached 0.88. Taking the Douyin platform as an example, this study applied the scale to measure the adolescent addiction level of using Douyin, including the following six items, specifically set as the following six questions: “I spent a lot of time thinking about the Douyin or plan to use Douyin” “I want more and more frequently swipe Douyin” “I swipe Douyin to escape from reality,” “I didn’t use less Douyin” “If I were banned from using Douyin, I will become anxious” “In daily life I overuse the Douyin, which has a negative effect on work and study”. They are named SY1, SY2, SY3, SY4, SY5, and SY6 to reflect the addiction of adolescents to the use of short video apps.

For the questionnaire mentioned above, we adopted the translation and retranslation (translation and back translation) steps to ensure that the translation maintains the original meaning of the scale. In the first stage, we selected several English graduate students to translate the original English questionnaire, and then invited two experts with experience of questionnaire design and investigation to review and discuss the translation. In the second stage, several translators were invited to return the Chinese scale into English. It can correct inaccurate translation or items that may deviate due to language habits. Finally, the translation will be submitted to experts for comparison and modification to determine the official Chinese version of the questionnaire.

## Part 1: Adolescent Addiction Resulting From Their Own Factors

### Correlation Between Gender and Addiction Degree

This study conducted a chi-square test on gender and addiction degree and obtained a *P* value of 0.001 (below the significance level 0.05), that is, gender has a significant effect on the addiction degree of the use of short video apps use among adolescents. Therefore, a cross-analysis was carried out as shown in Table 1. The participants included 51 males and 45 females. Among males, 30 would spend much time thinking about or planning to use Douyin, accounting for 58.8% of the total number of males; 18 held a neutral attitude (choosing the option “General”), accounting for 35.3% of the total number of males; and three responded that they would not spend much time thinking about or planning to use Douyin, accounting for 5.9% of the total number of males. Among females, 30 would spend much time thinking about or planning to use Douyin, accounting for 66.6% of the total number of females; six held a neutral attitude, accounting for 13.3% of the total number of females; and nine would not spend much time thinking about or planning to use Douyin, accounting for 20% of the total number of females.

**TABLE 1:** Cross-sectional analysis of gender and addiction degree.

		I spent plenty of time thinking about or planning to use Douyin			Total
		
		Take a positive attitude	Be neutral	Take a negative attitude	
Gender	Male	3	18	30	51
	Female	9	6	30	45
	Total	12	24	60	96

The above analysis indicated that regarding whether to spend much time thinking about or planning to use Douyin, there was few difference between males and females who spent a lot of time thinking or planning to use Douyin (choosing “Agree” or “Strongly agree”), but three men did not spend a lot of time thinking about or planning to use Douyin (choosing “disagree” or “strongly disagree”), 5.9% of the male population; Nine women did not spend a lot of time thinking about or planning to use Douyin (choosing “disagree” or “strongly disagree”), 20% of the total number of the female population, so males were more likely to spend much time thinking about or planning to use Douyin than females. In addition, the percentage of the males who held a neutral attitude was also higher, which may lie in that they usually used Douyin but did not consider themselves addicted and they had thought about or planned to use Douyin but did not pay much attention to it. To conclude, among adolescents, compared with females, males were more likely to spend much time using short video apps, that is, male adolescents were more addicted to the use of short video apps.

### Correlation Between Age and Addiction Degree

According to the World Health Organization and the United Nations General Assembly, the age ranging from 15 to 25 is defined as the adolescence. In this study, the participants were divided into three groups based on their age and ensure its distribution of 15–25. From the demographic variable data of the respondents, in terms of age, 28.1% are aged 15–17, while 40.6% are aged 18–21, and 31.3% are aged 22–25. Specifically, 27 were in the group of 14–17 years old, 39 were in the group of 18–21 years old, and 30 were in the group of 22–25 years old. It can be seen that the age distribution of respondents is reasonable, which ensures the randomness and scientificity of the questionnaire issuance in this study and can be tested and analyzed. A chi-square test was conducted on correlation between age and addiction degree, and a *P* value of 0.008 was obtained (below the significant level 0.05), that is, the age has a significant effect on the degree of addiction to the use of short video apps among adolescents. Moreover, a cross-analysis was carried out as shown in Table 2. Among the participants aged 14–17, 15 failed to reduce their use of Douyin (choosing “Agree” or “Strongly agree”), accounting for 55.6% of the total in this group; 12 were neutral to the use of Douyin (choosing “General”), accounting for 44.4% of the total in this group; and none succeeded in reducing their use of Douyin (choosing “Disagree” or “Strongly disagree”). Among those aged 18–21, 27 failed to reduce their use of Douyin, accounting for 69.2% of the total in this group; 6 were neutral, accounting for 15.4% of the total in this group; and six succeeded in reducing their use of Douyin, accounting for 15.4% of the total in this group. Among those aged 22–25, 21 failed to reduce their use of Douyin, accounting for 70.0% of the total in this group; three were neutral, accounting for 10% of the total in this group; and six succeeded in reducing their use of Douyin, accounting for 20% of the total in this group. The above data show that among the subjects who failed to reduce their use of Douyin, the number of people aged 15–17 was 15, while the number of people aged 18–21 was 27. Among those who successfully reduced their use of the Douyin, there are six people aged 18–21 and 22–25. However, there is no population aged 15–17, indicating that the self-control of the population aged 15–17 is not enough. In the other two age groups, it has certain control ability, so some will succeed in reducing Douyin.

**TABLE 2:** Cross-sectional analysis of age group and addiction degree.

		I failed to reduce using Douyin			Total
		
		Take a positive attitude	Be neutral	Take a negative attitude	
Age Group	Aged 15–17	0	12	15	27
	Aged 18–21	6	6	27	39
	Aged 22–25	6	3	21	30
	Total	12	21	63	96

The above analysis revealed that with the increase of age, there was an increase in both proportions of the adolescents who increased or decreased their addiction to short video apps, which to some extent indicated the variability of this special group of adolescents. The adolescent addiction degree to the use of short videos has changed substantially, which also demonstrated the feasibility and necessity of providing guidance for adolescents.

### Correlation Between Family Environment and Addiction Degree

In this study, a chi-square test was conducted on the family members’ use of short video apps and the addiction degree, and a *P* value of 0.000 was obtained (below the significant level 0.01), that is, the family members’ duration of using short video apps has a significant effect on the adolescents’ addictive use of short video apps. Furthermore, a cross-analysis was carried out as shown in Table 3. There were 27 participants whose family members used Douyin for less than 1 h, and 12 of them failed to reduce their use of Douyin, accounting for 44.4% of the total in this group; nine were neutral (choosing “General”), accounting for 33.3% of the total in this group; and six succeeded in reducing the use of Douyin, accounting for 22.2% of the total in this group. There were nine participants whose family members used Douyin for 1–2.5 h, and three of them failed to reduce their use of Douyin, accounting for 33.3% of the total in this group; six held a neutral attitude, accounting for 66.7% of the total in this group; none succeeded in reducing the use of Douyin, accounting for 0% of the total in this group. There were 45 participants whose family members used Douyin for 2.5–4 h, and 33 of them failed to reduce their use of Douyin, accounting for 73.4% of the total in this group; nine were neutral, accounting for 20.0% of the total in this group; three succeeded in reducing the use of Douyin, accounting for 6.7% of the total in this group. There were 15 participants whose family members used Douyin for more than 4 h, of which 12 failed to reduce their use of Douyin, accounting for 80.0% of the total in this group; three held a neutral attitude, accounting for 20% of the total in this group; and none succeeded in reducing the use of Douyin.

**TABLE 3:** Cross-sectional analysis of family environment and addiction degree.

		I failed to reduce using Douyin			Total
		
		Take a positive attitude	Be neutral	Take a negative attitude	
Family Environment (Duration of using short video apps)	Within 1 h	6	9	12	27
	1–2.5 h	0	6	3	9
	2.5–4 h	3	9	33	45
	More than 4 h	0	3	12	15
	Total	9	27	60	96
					

The above analysis revealed that with the time increase of the family members’ use of Douyin, the proportion of the participants who failed to reduce their use of Douyin gradually increased in each group, which to some extent demonstrated that in the whole family environment, the duration of the use of Douyin by family members has a significant positive effect on the adolescent addiction to the use of short video apps.

## Part 2: Correlation Between the User Generated Content and Adolescent Addiction

### Internal Consistency

This study adopted Cronbach’s Alpha to measure the internal consistency of latent variables. The Cronbach’s Alpha of daily life boredom (RC), addictive use of short video apps (SY), and UGC perception (UG) was, respectively, 0.856, 0.908, and 0.901, all greater than 0.7 (Tavakol and Dennick, 2011; Cho and Kim, 2015; Taber, 2018), indicating that the theoretical model of this study had excellent internal consistency.

### Discriminant Validity

According to the definition of discriminant validity by Deng et al. (2014), the average extracted variation (AVE) should be greater than corresponding correlation values. The research data showed that the AVE of daily life boredom (RC), addictive use of short video apps (SY) and UGC perception (UG) was 0.882, 0.828, and 0.847, respectively. The correlation coefficient between daily life boredom (RC) and addictive use of SY was 0.650, the correlation coefficient between daily life boredom (RC) and UG was 0.631, and the correlation coefficient between addictive use of SY and UG was 0.747. Thereby, the AVE values were greater than other correlation coefficients, proving that the theoretical model in this study had strong discriminant validity.

### Confirmatory Factor Analysis

In this manuscript, UGC Perception, Boredom in Daily Life and Addiction were selected as measurement factors. UGC Perception included five questions, Boredom in Daily Life included 3 questions, and Addiction included six questions, as shown in Table 4. On this basis, a preliminary factor load analysis was conducted, and it was found (as shown in Table 5) that the total explanatory rate of factors with eigenvalues greater than 1 was 76.173%. It indicates that the extracted three factors could explain 76.173% of the total variance of the original variables and meet 70% of the basic conditions (Smith, 2002; Shlens, 2014). Therefore, these factors have good representativeness.

**TABLE 4:** Setting and source of factor items.

Constructs	Measurement items	Sources
UGC perception	UG1: How much is Douyin in your life?	Rigby et al., 2019
	UG2: To what extent do you forget your daily worries while swiping Douyin?	
	UG3: To what extent do you feel that the content of Douyin’s short videos is something you’re experiencing, rather than just something you’re watching?	
	UG4: To what extent do you feel like you’re swiping Douyin on your own terms?	
	UG5: When you stop swiping Douyin, to what extent do you want to swipe Douyin again?	
Boredom in daily life	RC1: Everyday life is not going as I planned	O’Brien and Cairns, 2015
	RC2: I often lose myself in daily life and feel directionless	
	RC3: In my daily life, I often get caught up in something and lose track of time	
Addiction	SY1: I spend a lot of time thinking about Or planning to use Douyin	Smith and Short, 2022
	SY2: I want to swipe Douyin more and more frequently	
	SY3: I use Douyin to escape from reality	
	SY4: I fail to reduce the use of Douyin	
	SY5: If I was banned from using Douyin, I would get anxious	
	SY6: I overuse Douyin in my daily life, which has a negative impact on my work and study	

**TABLE 5:** Total variance interpretation.

Component	Initial eigenvalues			Extraction sums of squared loading			Rotation sums of loading		
			
	Total	Percentage of variance %	Cumulative %	Total	Percentage of variance %	Cumulative %	Total	Percentage of variance %	Cumulative %
UG	7.974	56.960	56.960	7.974	56.960	56.960	4.197	29.976	29.976
SY	1.526	10.902	67.862	1.526	10.902	67.862	3.681	26.291	56.267
RC	1.164	8.311	76.173	1.164	8.311	76.173	2.787	19.906	76.173

*Extraction method: principal component analysis.*

**FIGURE 2:**
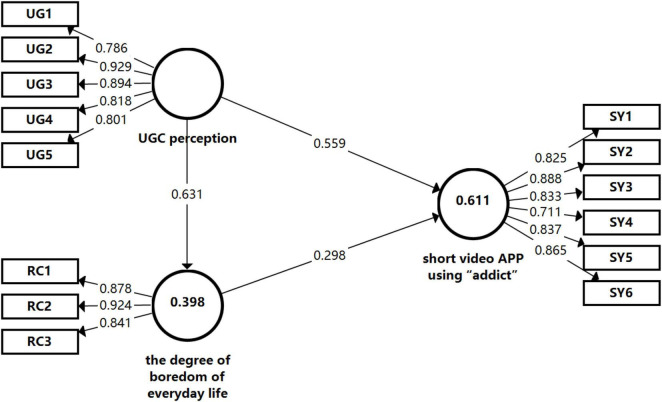
Structural model of adolescent addiction to short video apps.

In line with the threshold criteria suggested by related studies (Fornell and Larcker, 1981; Chernev and Blair, 2015; Kline, 2015), the load should be greater than 0.7, the average extracted variation (AVE) should be greater than 0.5, and the composite reliability (CR) should be greater than 0.7 (see Figure 2). The data showed that the respective load of the three items relating to the daily life boredom level (RC) was 0.878, 0.924, and 0.841, all greater than 0.7; the respective load of the six items relating to the addictive use of SY were 0.825, 0.888, 0.833, 0.711, 0.837, and 0.865, all greater than 0.7; the respective load of the five items relating to UG were 0.786, 0.929, 0.894, 0.818, and 0.801, all greater than 0.7, meeting the relevant criteria. And the AVE of RC, SY, and UG was respectively, 0.777, 0.686, and 0.718, all greater than 0.5, indicating that the theoretical model of this study had convergent validity. Besides, the CR of RC, SY and UG was respectively, 0.913, 0.929, and 0.927, all greater than 0.7, indicating that the theoretical model of this study had strong composite reliability. The above analysis suggested that the overall goodness of fit of the model was excellent, the internal latent relationship had a significant explanatory effect, the estimated effect was acceptable, and the reliability of each variable was consistent with the construct validity.

### Structural Equation Modeling and Mediating Effect Analysis

The Table 6 showed that the path coefficient between daily life boredom (RC) and addictive use of SY was 0.298 and the *p* value was 0.000, indicating that daily life boredom had a significant positive effect on addictive use of short video apps, thus Hypothesis 2 was verified; the path coefficient between UG and daily life boredom (RC) was 0.631 and the *P* value was 0.000, indicating that UGC perception had a significant positive effect on daily life boredom, thus Hypothesis 3 was verified; the path coefficient between UGC and addictive use of SY was 0.559 and the *P* value was 0.000, indicating that UGC perception had a significant positive effect on addictive use of short video apps, thus Hypothesis 1 was verified. In addition, the path coefficient between UGC perception and addictive use of short video apps under the mediation of degree of boredom in daily life was 0.188, and the *P* value was 0.000, less than the significance level 0.05, indicating that the mediating effect was significant and the degree of boredom in daily life had a significant mediating effect between UGC perception and addictive use of short video apps, thus Hypothesis 4 was verified.

**TABLE 6:** Path analysis.

	*T* value	*P* value	Path coefficient	Hypothesis
RC–>SY	4.158	0.000	0.298	H2 verified
UG–>RC	10.876	0.000	0.631	H3 verified
UG–>SY	6.131	0.000	0.559	H1 verified
UG–>RC–>SY	3.707	0.000	0.188	H4 verified

## Research Conclusion and Implications

### Conclusion

Based on the influence of the UGC on adolescents’ immersive experience, this study explored the contributing factors of the adolescents’ addiction to the use of short video apps through two sub-studies and reached the following conclusions:

Part 1 analyzed the individual factors contributing to the adolescent addiction, including gender, age and family environment, finding that males have a higher addiction to the use of short video apps, the adolescent addiction to the use of short video apps varies with their age, and the duration of the family members’ use of the short video apps has a significant positive effect on the adolescent addiction to the use of short video apps.

Part 2 applied SmartPLS to verify the theoretical model proposed in this study. The results showed that both UGC perception and daily life boredom has a significant positive effect on the adolescent addiction to the use of short video apps, indicating that as for adolescents, the aggravation of state boredom would drive them to use the Internet media, and the richness and variety of the UGC and the freshness it brings may stimulate the adolescents to keep using short video apps. Additionally, UGC perception also has a significant positive effect on the degree of boredom in daily life, indicating that the richness of short video content intensifies the individuals’ addiction to short videos to a certain degree and reduces their interest in the surrounding things in daily life. Therefore, the degree of boredom in daily life has a significant mediating effect between UGC perception and stimulus addiction.

### Theoretical Implication

This study analyzed the contributing factors of the adolescents’ addiction to the use of short video apps and established a theoretical model based on the influence of the UGC on the adolescents’ immersive experience. From a theoretical point of view, the findings in this study are of great significance. Previous research was prone to view flow experience as a subjective psychological feeling (Quinn, 2005; Marty-Dugas and Smilek, 2019), linked with cognitive and emotional states (Khalid and Iida, 2021). In light of the novelty of the UGC and the boredom of daily life, this study analyzed the flow experience of the adolescents in a more objective way under the stimulation of new and old things and provided a new understanding and thinking of flow theory for the academic community. Moreover, most previous studies explored immersive experience based on the view of space (Wang and Yi, 2020), believing that social media platforms provided a media environment and cultural soil, and produced videos perpendicular to different fields to mobilize all the senses and cognitive experience of the individuals (Zhang et al., 2020) and make them engaged in the created atmosphere to reach an immersive state (Yue, 2021; Ghermandi et al., 2022). Flow theory also emphasized that in the process of experience (Zatori et al., 2018), the overall psychological attention of users shall be captured by constructing the interactive relationship between the subject and the object (Alvarez-Milán et al., 2018). This study took a process perspective and explored the adolescents’ immersive experience based on the UGC, which has clarified the complementation between the process view and spatial view on the immersive experience and enriched the academic research on the causes of addiction to the use of short video apps in adolescents.

### Practical Implication

Although short video apps enrich the adolescent daily life to some extent, cater to the adolescent psychological and recreational needs and broaden their horizons, the adolescent addiction to the use of short video apps may cause a series of problems. In order to make the online life of the adolescents healthier and address the addiction to short video apps, the analysis of the causes of addiction to the use of short video apps among adolescents based on the UGC in this study is conducive to further understanding the situation of the use of short video apps and the process of becoming addicted in adolescents in the hope of providing new ideas and methods for the protection of the adolescents relating to the use of short video apps in the future.

The findings of this study suggest that the long-term use of short video apps by family members will aggravate the adolescents’ addiction to short videos. Therefore, family members should set a good example for the adolescents, guide them to actively explore the fun in daily life and face life with a curious and positive attitude to avoid their excessive dependence on short video apps. Furthermore, the proportion of the adolescents who are addicted to short video apps varies with the increase of their ages, which to a certain degree shows the variability of this special group and further expounds the feasibility and necessity of providing guidance for them.

### Limitations

There are some limitations in this study, which needs to be further explored. First, as the participants in this study were only selected from the adolescents in Nanchang City, the sample was not strongly dispersed and had quantitative limitation. Future studies can examine the population of all ages in various regions to improve and expand the sample data. Second, in the analysis of individual factors of the adolescents’ addiction, this study only analyzed three factors including gender, age group and family environment, whereas other potential factors may also have an impact on adolescents’ addictive exposure to short video apps, which may be further explored in the future research. Third, in the correlation analysis between family environment and addiction degree, the family environment factor discussed in this study was not comprehensive enough, and the blank area needs to be explored for more valuable information. Fourth, in the correlation analysis between age and addiction degree, the variability of this special group of adolescents was proved, and the degree of addiction to the use of short videos will change during the adolescent growing up, implying that it is feasible and necessary to give them appropriate guidance, nevertheless, this study does not design experiments to explore the specific ways and effects of such guidance which is worthy of future research. Fifthly, all scales used in this research are selected from mature scales, but the suitability of the model needs to be further considered. At the same time, because the selected scale is English and the research object is Chinese teenagers, there are some errors in the translation process, which will affect the research results to a certain extent.

## Data Availability Statement

The original contributions presented in the study are included in the article/supplementary material, further inquiries can be directed to the corresponding author.

## Ethics Statement

The studies involving human participants were reviewed and approved by East China Jiaotong University. The patients/participants provided their written informed consent to participate in this study. Written informed consent was obtained from the individual(s) for the publication of any potentially identifiable images or data included in this article.

## Author Contributions

LL contributed to the empirical work, analysis of the results, and wrote first draft of the manuscript. BG advised the hypotheses development and revised the manuscript. ML, BG, ZB, and ZL supported the total work of the LL. All authors discussed the results, commented on the manuscript, and approved the submitted version.

## Conflict of Interest

The authors declare that the research was conducted in the absence of any commercial or financial relationships that could be construed as a potential conflict of interest.

## Publisher’s Note

All claims expressed in this article are solely those of the authors and do not necessarily represent those of their affiliated organizations, or those of the publisher, the editors and the reviewers. Any product that may be evaluated in this article, or claim that may be made by its manufacturer, is not guaranteed or endorsed by the publisher.
